# Transcatheter arterial chemoembolization for lung malignant tumors

**DOI:** 10.3389/fonc.2025.1551644

**Published:** 2025-04-16

**Authors:** Fenxiang Zhang, Yujin Liu

**Affiliations:** Department of Interventional Oncology, Yueyang Hospital of Integrated Traditional Chinese and Western Medicine, Shanghai University of Traditional Chinese Medicine, Shanghai, China

**Keywords:** lung cancer, interventional radiology, pulmonary artery, bronchial artery, chemoembolization

## Abstract

Transcatheter arterial chemoembolization may enhance the local concentration and cytotoxicity of chemotherapeutic drugs and block the blood supply to the tumor, with an expectation to control the tumor, relieve hemoptysis, and prolong survival. This review will introduce the research progression of blood supply to lung cancer, discuss pulmonary artery chemoembolization (PACE) and bronchial artery chemoembolization (BACE) for lung cancer, including their indications and contraindications, operation techniques, efficacy assessments, combined treatments as well as their operating complications and the methods to prevent the adverse event. We will discuss the problems and challenges of percutaneous vascular intervention for lung cancer, such as the uncertainty of blood supply artery for lung cancer, the necessity of high-quality controlled studies, and the best choice for the indications. We hope to explore the direction of transcatheter arterial chemoembolization for lung cancer. The aim of this review is to provide a reference for the practice of chemoembolization by vascular interventional radiology for lung cancer.

## 1 Introduction

Lung cancer is a common malignant tumor that typically originates from the bronchial epithelial cells (1). There are two main subtypes of lung cancer: non-small-cell lung cancer (NSCLC) and small-cell lung cancer (SCLC). NSCLC is the more common subtype, accounting for about 80 to 85 percent of all cases, while SCLC represents only 15 to 20 percent of cases (2).

Approximately 30% of lung cancer patients are eligible for surgery. While traditional treatments such as chemotherapy and radiation can be effective, they come with significant side effects, particularly for older patients (3). Molecularly targeted therapies have shown positive results in less than half of lung adenocarcinoma cases and often face challenges due to resistance. Additionally, immunotherapy has been effective for only a small subset of lung cancer patients (4).

Arterial chemoembolization techniques, such as PACE (Pulmonary Artery Chemoembolization) and BACE (Bronchial Artery Chemoembolization), are used to treat lung cancer by targeting the blood vessels that supply the tumor. Recent advances in imaging, including multislice spiral CT and DSA (digital subtraction angiography), have shown that the pulmonary artery is not typically involved in the blood supply of lung cancer (5, 6). Instead, the bronchial artery, which is part of the body’s general circulatory system, plays a central role in supplying blood to lung cancer. As a result, BACE is increasingly replacing PACE in the treatment of lung cancer and has been more frequently reported in the literature. This article reviews the development of PACE and BACE as treatment options for lung cancer.

## 2 Blood supply of lung cancer

The question of how lung tumors receive their blood supply has long been debated, particularly regarding the roles of the bronchial and pulmonary arteries. Early studies suggested that both arteries contributed to the tumor’s blood flow (7). However, more recent imaging techniques, such as CT angiography and DSA, along with preclinical models, increasingly support the idea that the bronchial arteries are the primary source of blood for lung cancer (8–10). **Table 1** summarizes key preclinical and clinical findings on this topic.

**Table 1:** Summary of key studies on lung cancer blood supply.

Study(Year)	Model/Patients	Key Findings	Clinical Implication
Xiao et al. (1997) (8)	32 lung cancer patients	Bronchial arteries sole blood source; no pulmonary artery involvement	Supports BACE over PACE
Sun et al. (2021) (9)	Canine lung tumor model	Tumors primarily supplied by bronchial arteries	Validates BACE in animal models
Deng et al. (2020) (10)	Murine lung adenocarcinoma	84% of early-stage tumors supplied by pulmonary arteries	Suggests role of PACE in early lesions
Li et al. (2022) (11)	54 advanced NSCLC cases	Dual bronchial/pulmonary supply in peripheral tumors; BACE effective	Highlights need for individualizedTACE

**Figure 1:**
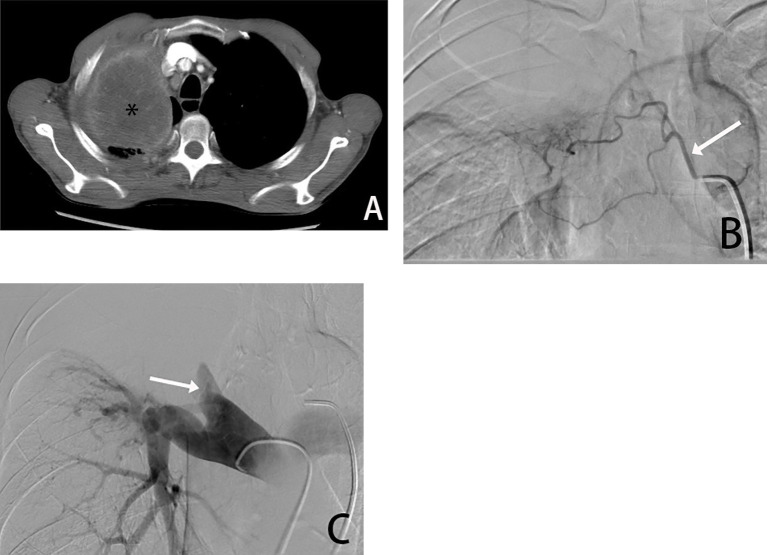
Male, 66 years old, tumor found in the right upper lung due to chest pain, biopsy pathology diagnosed squamous cell carcinoma. **(A)** is a CT transverse image showing a right upper lung tumor(*). **(B)** shows a right bronchial arteriogram (→) showing that the right lung tumor is supplied by the right bronchial artery, and **(C)** is a pulmonary arteriogram showing compressive violation of the right upper pulmonary artery, which is not involved in the blood supply.

**Figure 2:**
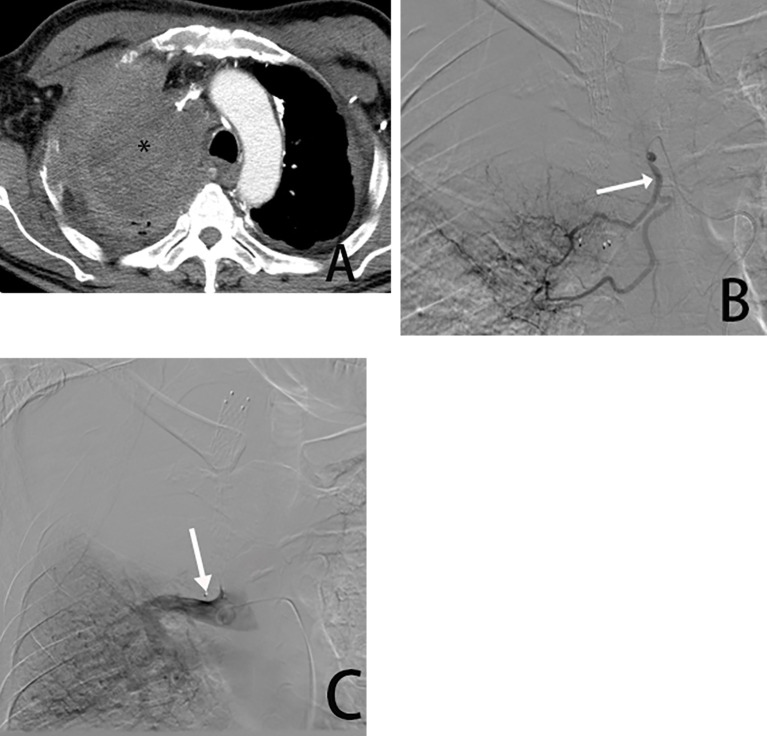
Male, 54 years old, tumor was found in the right upper lung. **(A)** is a CT cross-sectional view showing the right upper lung tumor (*). **(B)** is a right bronchial arteriogram (→) showing that the right lung tumor is supplied by the bronchial artery. **(C)** is a pulmonary arteriogram showing that the pulmonary artery was not involved in the blood supply(→) and was occluded due to compression by the tumor encompassing it.

**Figure 3:**
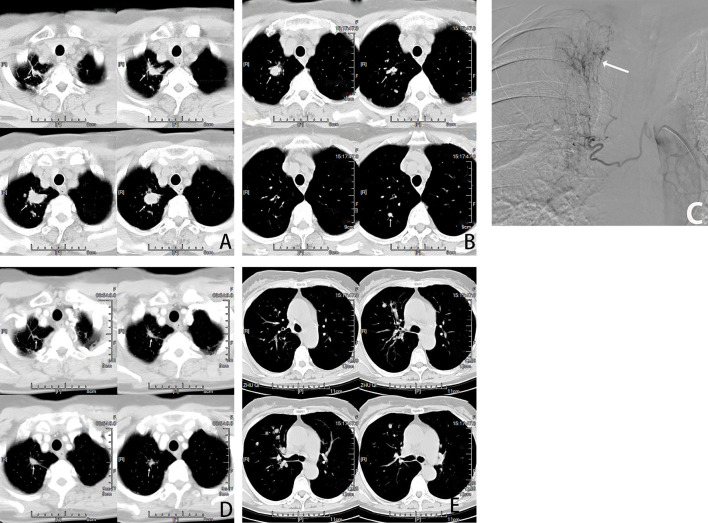
Female, 66 years old, lung adenocarcinoma treated with molecularly targeted drug therapy (gefitinib) for 2 years after tumor progression with multiple nodal metastases in the right lung. **(A, B)** are CT cross-sectional images of the patient, showing Multiple nodules(→) in the right lung. **(C)** is a right bronchial arteriogram showing that the right lung tumor(→) is supplied by the right bronchial artery. BACE chemotherapeutic agent: pemetrexed 800mg + carboplatin 300mg and gelatin sponge particles. **(D, E)** show that most of the nodules(→) in the lungs disappeared and shrunk after two courses of BACE, and PR was assessed.

**Figure 4:**
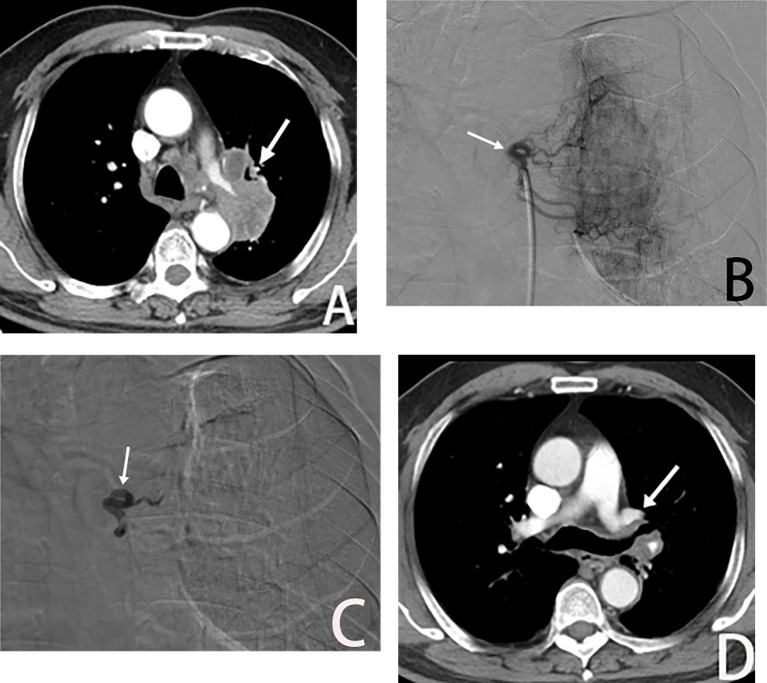
Male, 69 years old, postoperative recurrence of lung adenocarcinoma. **(A)** The left bronchial artery supplying the left lung tumor (→), BACE was performed with a quality regimen: 200 mg of albumin paclitaxel, 300 mg of carboplatin, and gelatin sponge particles. **(B, C)** show left bronchial artery arteriogram and embolized (→) on DSA. **(D)** is CT scan showed significant tumor shrinkage (→), and PR was assessed after 3 weeks of BACE.

**Figure 5:**
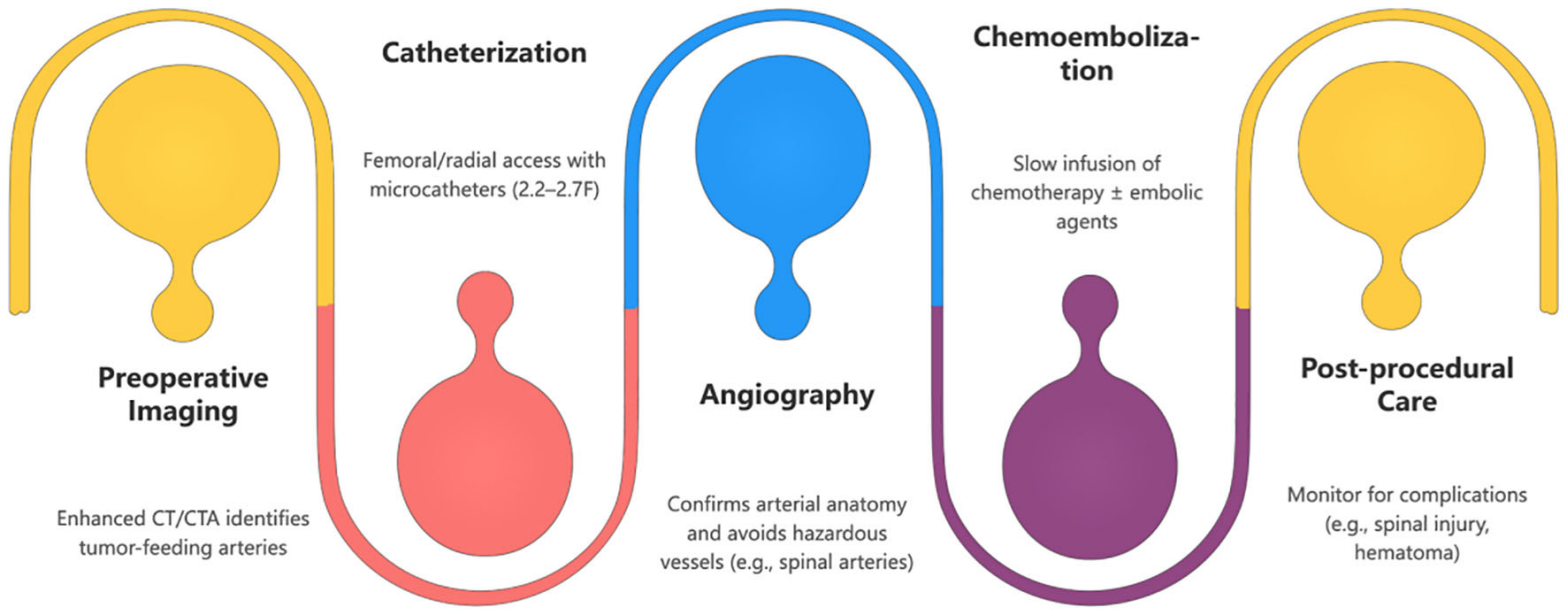
The diagram illustrates the key stages of BAI/BACE. Each stage is clearly marked for easy understanding. Suitable for patient education, medical training, or clinical reference.

According to the majority of current research, the bronchial artery is now recognized as the primary blood vessel supplying lung cancer, while the pulmonary artery plays a secondary role. Our findings, shown in **Figures 1** and **2**, confirm that the bronchial artery provides the main blood supply to central lung cancer in most cases, while the pulmonary artery does not contribute significantly. However, further clinical and basic research is needed to determine whether the pulmonary artery plays a role in the blood supply of peripheral lung cancer, particularly in smaller peripheral tumors. Our observations, as seen in **Figures 3** and **4**, show that both lung metastases and peripheral lung cancer clearly receive blood from the bronchial artery, with the pulmonary artery not being involved in the blood supply (**Figure 5**). For these cases, BACE has shown satisfactory efficacy.

## 3 PACE for lung cancer

Pulmonary art chemoembedding (PACE) for lung cancer was first attempted in the 1950s, but it did not gain widespread acceptance due to technological limitations and safety concerns (11). However, after the 1980s, the procedure gained renewed attention because it has proven to be effective in slowing the progression of lung cancer, improving patient survival, and enhancing their quality of life (12). Studies have shown that PACE can slow tumor growth in patients with locally advanced or metastatic non-small-cell lung cancer (NSCLC), increase median survival times, relieve symptoms, and improve overall quality of life.

## 4 BACE for lung cancer

### 4.1 The emergence and development of BACE

BACE (Bronchial artery chemoembolization) is a treatment that delivers chemotherapy directly through the bronchial artery while using embolic particles to block blood flow to the tumor. This approach helps to destroy the tumor cells and cause ischemic necrosis, minimizing systemic side effects and preserving immune function. This method is especially beneficial for advanced lung cancer patients who experience symptoms like coughing up blood (hemoptysis) and shortness of breath (dyspnea). Over time, BACE has evolved through several stages: perfusion chemotherapy, embolization, combined therapies, and the use of drug-eluting microspheres (13).

In the perfusion chemotherapy stage, Japanese researchers first began using bronchial artery chemotherapy infusion (BAI) to treat lung cancer in the 1970s (14). Although there was some success, issues like poor drug distribution, inadequate doses, and significant side effects limited its effectiveness.

In the embolization stage, researchers introduced bronchial artery embolization (BAE) combined with chemotherapy. This method involved using embolic agents like thrombin and gelatin particles, which helped reduce symptoms such as hemoptysis and tumor size (15).

In the drug-eluting microspheres stage, researchers like Xu Yuhua et al. (16) pioneered the use of drug-eluting microspheres in chemoembolization for lung cancer. These polymer-based microspheres, such as DC Bead, Hepersphere, and CalliSpheres, have dual functions. They can carry chemotherapy drugs for gradual, controlled release while simultaneously blocking blood flow to the tumor (17).

In the combination therapy stage, researchers are exploring how to integrate BACE with other treatments, such as brachytherapy (18), molecular targeted therapies, immunotherapy, and radiation therapy. This approach has shown promise in improving survival rates and the quality of life for lung cancer patients (19).

### 4.2 Anatomy and clinical significance of bronchial arteries

Bronchial arteries play a vital role in lung vascular supply, delivering oxygenated blood to bronchial walls, lung parenchyma, pulmonary lymphoid tissues, and the visceral pleura. They contribute to lung defense, metabolism, and gas exchange. Individual anatomical variations in bronchial arteries, such as their number, origin, distribution, and course, exist (20). Familiarity with bronchial artery anatomy is crucial for recognizing arterial lesions, enabling differential diagnosis, and guiding interventional therapies.

Almeida et al. (21) categorized the anatomical origin of bronchial arteries into two types: normal (orthotopic) and ectopic, based on their connection to the descending thoracic aorta.

Bronchial arteries of normal origin typically arise from the descending thoracic aorta near the T5-T6 vertebral plane, located 1-2 cm above and below the tracheal bulge (21). The right bronchial artery primarily originates from the intercostal bronchial trunk or the aorta, while the left bronchial artery mainly originates from the anterior or lateral wall of the aorta, with its primary source being the thoracic aorta or its branches, including intercostal and subclavian arteries.

Common variants in bronchial artery origins, as classified by Ittrich (22) and Mansur (23), fall into four types: (1) type 1, two left bronchial arteries arise from the descending thoracic aorta and one right bronchial artery originates from the intercostal artery (ICBT); (2) type 2, one right bronchial artery originating from the intercostal artery and one left bronchial artery from the descending thoracic aorta; (3) type 3, two right bronchial arteries having distinct origins, one from the intercostal artery and the other from the descending thoracic aorta, and two left bronchial arteries both originating from the descending thoracic aorta; (4) type 4, one left bronchial artery originates from the descending thoracic aorta, and two right bronchial arteries have separate origins, one from the intercostal artery and the other from the descending thoracic aorta.

Additionally, bronchial arteries with ectopic origins, arising from vessels like the aortic arch and subclavian artery, can be observed. Identification of ectopic bronchial arteries can be based on their proximity to the corresponding bronchus. According to Stoll’s literature (24), seven distinct types of ectopic bronchial artery origins are documented, including the subclavian, thoracic, pericardial diaphragmatic, innominate, thyroid carotid, subphrenic, and abdominal aorta. Mansur et al.’s research (23) also mentions the rare possibility of bronchial arteries originating from the left gastric artery. These arteries may either have juxtaposed origins or emerge from a common trunk. In summary, bronchial arteries exhibit diverse origins.

### 4.3 Operating technique

The BACE procedure involves superselective catheterization of the bronchial arteries, followed by chemotherapy infusion (e.g., cisplatin or carboplatin) and embolization with materials like gelatin sponges or drug-eluting microspheres (12). The flowchart below illustrates the steps involved in this process.

### 4.4 Indications and contraindications

#### 4.4.1 Indications

BACE is an important treatment option for patients with advanced lung cancer who are not candidates for standard therapies. In the literature, BACE is primarily used for patients with intrathoracic tumors, particularly those with stage IIIb or more advanced inoperable non-small-cell lung cancer (NSCLC) and small-cell lung cancer (SCLC). For patients with extrapulmonary metastases, BACE can be combined with other treatments to address intrapulmonary lesions. Possible indications include:

1. Failure, progression, or recurrence of stage IIIb or more advanced NSCLC and SCLC after standard treatments (radiotherapy, targeted therapy, immunotherapy).
2. Patients unable to tolerate or declining standard treatments.
3. Combination therapy with standard treatment.
4. Adjuvant therapy following surgical resection recurrence.
5. Emergency management for complications like hemoptysis.
6. Prophylactic hemostatic treatment before endoscopic treatment of endotracheal lesions.
7. Lung cancer in conjunction with airway stenosis or pulmonary atelectasis (25).

While the Lung Cancer Diagnostic and Treatment Guidelines (26) suggest that patients with stage IV NSCLC and an Eastern Cooperative Oncology Group (ECOG) Physical Status Score >2 may not typically benefit from systemic chemotherapy, considering that BACE mainly local efficacy with minimal systemic side effects, it is advisable to actively assess the potential of BACE while implementing optimal supportive care. By judiciously reducing the chemotherapy agent dosage, it remains possible to effectively manage the tumor, alleviate symptoms, enhance the quality of life, and capitalize on the benefits of BACE.

#### 4.4.2 Contraindications

Before considering BACE for lung cancer treatment, it is essential to rule out the following contraindications or relative contraindications:

1. Blood analysis indicated WBC < 3.0×10⁹/L, Neutrophils < 1.5×10⁹/L, RBC < 2.0×10¹²/L, Hb < 80 g/L, and Platelets < 50×10⁹/L.
2. Severe bleeding tendency and coagulation dysfunction, not correctable within a short timeframe (prothrombin time >18 s, prothrombin activity <40%).
3. Severe pulmonary fibrosis, pulmonary hypertension, or reduced pulmonary circulation due to various reasons.
4. Infectious or radiation-related inflammation around the lesion, uncontrolled skin infection at the puncture site, systemic infection, and fever >38.5°C.
5. Severe hepatic, renal, cardiac, pulmonary, and cerebral insufficiency, severe anemia, dehydration, and significant nutritional metabolism disturbances that cannot be corrected or improved within a short period.
6. Uncontrolled malignant pleural or pericardial effusion.
7. Patients with extensive metastatic tumors and an expected survival of less than 3 months.
8. Allergy to iodine-containing contrast agents, inability to lie supine, inability to cooperate with puncture, intubation, and contrast procedures, and patients with a history of psychosis.
9. Intraoperative imaging reveals an inability to achieve super-selective cannulation to completely avoid spinal arteries and other hazardous vessels.
10. Inability to achieve selective bronchial artery cannulation following preoperative assessment, especially after descending thoracic aortic overlay stent implantation.

### 4.5 Principles of selection

Treatment plans are tailored to each patient’s pathology, medical history, and lab results. Chemotherapy agents are selected based on tumor characteristics and pharmacokinetics, with a focus on combining drugs that have complementary effects. In arterial perfusion chemotherapy, drugs that are concentration-dependent and cell-cycle nonspecific are preferred to enhance cytotoxicity, while minimizing the use of cell-cycle specific drugs (27–29).

Systemic chemotherapy remains the primary treatment for lung cancer, as outlined in the 2023 National Health guidelines, which provide recommended first, second, and third-line drug regimens for advanced stages (26). The initial treatment for advanced (NSCLC) typically involves platinum-based combinations, such as cisplatin or carboplatin (sometimes nedaplatin), along with agents like vincristine, gemcitabine, or paclitaxel. Dosages are based on body surface area, with carboplatin targeted to an area under the curve (AUC) of 5-6. Treatment usually involves 4-6 cycles, each lasting 21 days. Some drugs, such as vincristine and gemcitabine, may be administered on day 8, and dosages are adjusted for intravenous use following BAI/BACE (30–34). **Table 2** outlines the standard BACE chemotherapy protocols, including drugs, mechanisms, dosages, and embolic agents.

**Table 2:** Chemotherapeutic agents.

	Mechanism	Dosage (BSA-based)	Embolic Agent
Cisplatin	DNA crosslinking	75–100 mg/m²	Gelatin sponge
Carboplatin	Alkylation	AUC 5–6	Drug-eluting microspheres
Pemetrexed	Antimetabolite	500 mg/m²	Polyvinyl alcohol particles
Albumin-bound paclitaxel	Microtubule inhibition	260 mg/m²	–

### 4.6 Evaluation of efficacy

The effectiveness of BACE for lung cancer is assessed using the RECIST or mRECIST criteria, which classify responses as complete response (CR), partial response (PR), stable disease (SD), or progressive disease (PD). Key metrics such as overall response rate (ORR) and disease control rate (DCR) are also used. Additionally, evaluations consider tumor markers, patient symptoms, quality of life, and side effects. Long-term outcomes, such as progression-free survival (PFS) and overall survival (OS), help guide adjustments in treatment strategies during the course of patient care.

### 4.7 Combination therapy

The management of lung cancer can be enhanced by combining BACE with other treatments such as surgery, radiotherapy, particle implantation, and targeted therapy. **Table 3** summarizes the outcomes from key BACE studies.

**Table 3:** Efficacy of BACE in lung cancer.

Study (Year)	Patients (n)	ORR (%)	Median OS (months)	Key CombinationTherapies
Huang et al. (2008) (35)	127	59.2	14.5	BACE + systemic chemotherapy
Jiang et al. (2022) (36)	45	68.9	18.2	BACE + immunotherapy
Xie et al. (2022) (27)	72	73.6	21.0	BACE + ¹²⁵I seed implantation

The combined use of BACE and immunotherapy offers several distinct advantages. This approach allows for localized, high-concentration chemotherapy, which triggers the release of antigens and inflammatory factors from tumor cells. It activates the immune system, enhancing the efficacy of immunotherapy, while reducing the required dosage and frequency of immune checkpoint inhibitors. This combination also helps mitigate immune-related adverse reactions (37, 38).

Additionally, BACE plays a crucial role in the treatment of postoperative lung cancer recurrence (**Figure 4**). In cases of lung adenocarcinoma recurrence after surgery, where no driver gene mutations are present and tumor progression persists despite adjuvant chemotherapy, BACE therapy has led to partial responses (PR).

### 4.8 Prevention and manegment of complications

Compared to systemic chemotherapy, BACE offers benefits such as targeted efficacy, improved quality of life, and reduced systemic toxicity, resulting in fewer side effects like nausea and myelosuppression. However, it is important to be aware of potential severe complications, including spinal cord injury and mediastinal fistula.

To reduce the risk of rare but severe paraplegia due to spinal cord injury during BACE, careful angiography with diluted nonionic contrast (39–41), superselective cannulation of relevant arteries, and pre-perfusion lidocaine testing to check for signs of spinal anesthesia are crucial. Continuous monitoring of sensory and motor functions during and after the procedure is essential. If spinal injury occurs, immediate cessation of treatment and symptomatic management, including the use of glucocorticoids and vasodilators, should be implemented (25).

Rare complications like tracheoesophageal injuries and acute esophagitis during BACE require identifying the esophageal artery branches to avoid them before treatment (42). Management of these complications is typically symptomatic, and may involve fasting. Esophageal bronchial fistula formation, which occurs due to drug-induced esophageal damage, presents as increased coughing and is alleviated by consuming solid food. The primary treatment for this condition is sealing the esophagus with a stent, with prevention focusing on detailed imaging, controlled drug dosing, and vigilant symptom monitoring (42).

Bleeding complications can arise from various sources, including puncture site bleeding, hematoma, and pseudoaneurysm (37). Arterial injuries, such as bleeding from renal artery damage, thoracic aortic endometrial tears, bronchial artery dissecting aneurysms, and rupture hemorrhages, can occur if fluoroscopy is not used in a timely manner or if there is rough handling during the procedure (38, 43).

### 4.9 Combining targeted therapy and immunotherapy

The synergy between immunotherapy, targeted therapy, and radiation therapy shows significant promise in cancer treatment. BACE-induced tumor antigen release enhances the effectiveness of PD-1/PD-L1 inhibitors (44–47). In targeted therapy, using EGFR inhibitors like gefitinib alongside BACE improves outcomes in patients with mutation-positive (NSCLC) (3). Moreover, BACE reduces tumor hypoxia, which enhances the effects of radiotherapy. By combining these treatment strategies, more personalized and effective options are available for cancer patients (4, 48).

## 5 Prospects

Percutaneous vascular interventions have shown potential in treating lung cancer, but there are challenges that still need to be explored. Existing research is limited, especially regarding systemic chemotherapy, and there is a need for high-quality studies, particularly randomized controlled trials, to assess the impact of these interventions on patient outcomes. Additionally, it is important to better understand how vascular interventions work alongside radiotherapy and immunotherapy to provide a more comprehensive treatment approach
